# UHPLC-ESI-QTOF-MS/MS Profiling of Phytochemicals from Araticum Fruit (*Annona crassiflora* Mart.) and Its Antioxidant Activity

**DOI:** 10.3390/foods12183456

**Published:** 2023-09-16

**Authors:** Henrique Silvano Arruda, Célio Fernando Figueiredo Angolini, Marcos Nogueira Eberlin, Glaucia Maria Pastore, Mario Roberto Marostica Junior

**Affiliations:** 1 Department of Food Science and Nutrition, School of Food Engineering, University of Campinas, Rua Monteiro Lobato 80, Campinas 13083-862, São Paulo, Brazil; glaupast@unicamp.br (G.M.P.); mmarosti@unicamp.br (M.R.M.J.); 2 Institute of Chemistry, University of Campinas, Rua Josué de Castro s/n, Campinas 13083-970, São Paulo, Brazil; celio.fernando@ufabc.edu.br (C.F.F.A.); marcos.eberlin@mackenzie.br (M.N.E.); 3 Center for Natural and Human Sciences, Federal University of ABC, Avenida dos Estados 5001, Santo André 09210-580, São Paulo, Brazil; 4 MackMass Laboratory for Mass Spectrometry, School of Engineering, PPGEMN & Mackenzie Institute of Research in Graphene and Nanotechnologies, Mackenzie Presbyterian University, Rua da Consolação 896, São Paulo 01302-907, São Paulo, Brazil

**Keywords:** marolo, Cerrado fruit, Brazilian biodiversity, secondary metabolites, phenolic compounds, flavonoids, annonaceous acetogenins, biological properties, plant defense, human health

## Abstract

Araticum is a native species of the Brazilian Cerrado with a high potential for exploitation. Several studies have stated that araticum is a rich source of phytochemicals with multifaceted biological actions. However, little information is available regarding the characterization of phytochemicals found in the pulp of this fruit. In this context, this study aimed to carry out a comprehensive characterization of phytochemicals present in the araticum pulp using ultra-high-performance liquid chromatography coupled to a quadrupole time-of-flight mass spectrometer (UHPLC-ESI-QTOF-MS/MS). The antioxidant potential of araticum pulp was also evaluated. UHPLC-ESI-QTOF-MS/MS profiling of the phytochemicals allowed for the identification and annotation of 139 phytochemicals, including organic acids, jasmonates, iridoids, phenolic compounds, alkaloids, annonaceous acetogenins, fatty acid derivatives, and other compounds. Among them, 116 compounds have been found for the first time in araticum pulp. Phenolic compounds and their derivatives represented about 59% of the phytochemicals identified in the extract. Moreover, araticum pulp showed high total phenolic compound content and antioxidant activity. The majority of identified phytochemicals have been associated with key roles in the plant’s defense mechanisms against biotic and abiotic stress factors in the Cerrado environment. Furthermore, many of these phytochemicals found in the araticum pulp are already widely recognized for their beneficial effects on human health. Our findings showed that the araticum fruit contains different classes of phytochemicals that exert various biological activities, both in the plant itself and in humans.

## 1. Introduction

Brazil is home to one of the world’s greatest biodiversities, accounting for approximately 15–20% of global biological diversity, and it is the second country in terms of endemic species, surpassed only by Indonesia. The incredible Brazilian biodiversity is due to the presence of a large number of ecological biomes within its territory. Brazil is covered by six terrestrial biomes: the Amazon rainforest (tropical rainforest), Cerrado (savanna), Pantanal (marshlands), Atlantic Forest (subtropical forest), Pampa (lowlands), and Caatinga (semi-arid). Additionally, different distinct microbiomes are formed in coastal and marine regions. Currently, two of these biomes (the Cerrado and the Atlantic Forest) are considered biodiversity hotspots [1,2]. Thus, these unique characteristics of the Brazilian territory offer a wide range of possibilities for exploring and discovering nutritional and functional plants and fruits.

**Figure 1:**
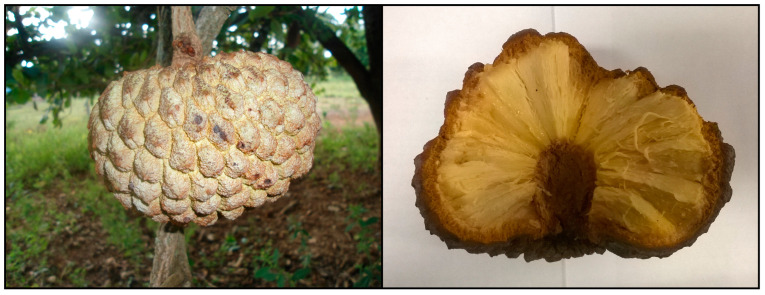
Araticum fruit (*Annona crassiflora* Mart.) used in the present study (detail of the fruit cross-section showing the cone-shaped buds (carpels) that compose the fruit pulp). Pictures taken by Henrique Silvano Arruda.

The Cerrado is the second-largest biome in Brazil, surpassed only by the Amazon rainforest, occupying approximately 2 million km^2^, which represents almost a quarter of the country’s total land area [3]. This biome is among the 25 most biodiverse sites in the world and is the richest tropical savanna on the planet, containing about 5% of all global diversity [4]. The Cerrado is home to 30% of the diverse living organisms identified in Brazil, including 12,356 naturally occurring species (e.g., herbaceous plants, shrubs, trees, and vines), of which 11,627 species are native and approximately 44% are endemic [4,5]. The vegetation of the Brazilian Cerrado is capable of withstanding extreme environmental conditions, including low water availability and high temperatures for long periods of the year, a high incidence of UV radiation, frequent wildfires, nutrient-poor soil, and recurrent attacks by insects and pathogenic microorganisms. Consequently, these plants have developed a series of adaptations throughout their evolutionary process to resist the oxidative stress occurring under these conditions, among which an upregulation in the synthesis of bioactive phytochemicals stands out. As a result, these species have great potential for use in various industrial sectors, with over 220 plant species already being employed in the formulation of food and medicines [4]. Although native plants of the Brazilian Cerrado present great potential for economic and technological exploration, many species remain unknown and/or unexplored, among which we can highlight the araticum (*Annona crassiflora* Mart.) (Figure 1).

The araticum, also known as marolo in some regions, is a native and endemic fruit of the Brazilian Cerrado widely used in regional cuisine and folk medicine. This fruit is highly appreciated by inhabitants of the Cerrado region due to its attractive color, intense flavor, and singular aroma. It is consumed in both fresh and processed products (e.g., ice creams, juices, jams, jellies, and popsicles, among others) [6]. Furthermore, this fruit has been used for centuries in local and traditional medicine as a tonic and astringent and for the treatment of pain and rheumatism [6]. Recent studies have shown that araticum pulp exerts various bioactivities, including antioxidant [7,8,9], anti-inflammatory [10], antibacterial [11,12], anti-Alzheimer’s [13], and anticancer effects [14]. These effects have been attributed to the presence of different bioactive phytochemicals that can be found in araticum pulp. Some recent studies have made efforts to characterize the bioactive phytochemicals present in araticum pulp, with the most frequently reported compounds belonging to the classes of phenolic compounds, alkaloids, annonaceous acetogenins, and carotenoids [6]. However, the number of studies with this approach is low, and the characterization data are often only partial and/or focused on a specific class of phytochemicals relevant to the work in question, particularly phenolic compounds. Therefore, the present study aimed to comprehensively characterize the profile of phytochemicals present in araticum pulp using ultra-high-performance liquid chromatography coupled to a quadrupole time-of-flight mass spectrometer (UHPLC-ESI-QTOF-MS/MS) and evaluate its antioxidant potential through different assays. Furthermore, we conduct here for the first time an integrated discussion regarding the key role of the identified phytochemicals in the development, adaptation, and resistance of the plant to its native environment (Cerrado biome), as well as the potential beneficial effects on human health and well-being associated with the consumption of these phytochemicals.

## 2. Materials and Methods

### 2.1. Chemicals and Reagents

Gallic acid, 6-hydroxy-2,5,7,8-tetramethylchroman-2-carboxylic acid (Trolox), 2,2′-azinobis-(3-ethylbenzothiazoline-6-sulfonic acid)-diammonium salt (ABTS), 2,2′-azobis-(2-methylamidinopropane)-dihydrochloride (AAPH), methanol, acetonitrile, and formic acid-grade HPLC were obtained from Sigma-Aldrich Chemical Co.^®^ (St. Louis, MO, USA). All other solvents and reagents used in this study (Folin–Ciocalteu reagent, sodium carbonate, potassium persulfate, potassium phosphate monobasic, potassium phosphate dibasic, fluorescein, methanol, and acetone) were at least of analytical grade. The ultrapure water to prepare the solutions was obtained from a Milli-Q water purification system (Millipore, Bedford, MA, USA).

### 2.2. Plant Material and Sample Preparation

Six fully ripe and morphologically perfect araticum fruits (totaling approximately 10 kg) were collected after naturally falling from the trees in natural areas of the Brazilian Cerrado located in the city of Carmo do Paranaíba (19°00′03″ south latitude, 46°18′58″ west longitude, and 1061 m altitude), Minas Gerais, Brazil. The fruits were washed with tap water and then manually peeled and pulped. The pulp was freeze-dried (LIOTOP^®^, model L101, São Carlos, Brazil), ground using a knife mill (Marconi^®^, model MA340, Piracicaba, Brazil), and stored at −20 °C until analysis.

A voucher specimen (UEC 197249) was deposited in the Herbarium of the Institute of Biology of the University of Campinas, Brazil (Herbarium UEC). The Genetic Heritage Management Board (CGen), under number A437549, following Law n° 13.123/2015 and its regulations, regimented the activity of accessing genetic heritage.

### 2.3. Extraction Procedure

Ultrasound-assisted solid-liquid extraction (USLE) was applied in this study for extracting phytochemicals from araticum pulp. This procedure was chosen based on the good results previously attained by our research group in obtaining phytochemical-rich extracts from araticum peel [15]. A 13 mm diameter probe with a nominal input power of 600 W and a constant frequency of 19 kHz (Unique, Disruptor, 800 W, Indaiatuba, Brazil) was employed for sample sonication. A mixture of methanol–acetone–water (7:7:6, *v*/*v*/*v*) was selected as the extractor solvent because this solvent combination has been considered an excellent option for extracting soluble phenolic compounds from plant matrices [8,16]. Thus, USLE was performed by mixing 1.25 g of freeze-dried araticum pulp with 25 mL of extractor solvent in a 50 mL centrifuge tube. The ultrasonic probe was inserted directly into the tube to provide direct contact with the sample, dipping 20 mm into the extraction mixture. The sample was subjected to sonication at a nominal input power of 600 W for 5 min, resulting in an energy density of 7.2 kJ/mL. To protect thermolabile phytochemicals, an ice bath was used to prevent overheating of the sample during the extraction procedure. The temperature of the sample measured at the system outlet was 27.6 °C. After extraction, the extract was centrifuged at 4000× *g* for 11 min at 5 °C (Hettich Zentrifugen, model Rotanta 460R, Tuttlingen, Germany). The supernatant was collected, filtered through a 0.22 µm PTFE syringe filter, and stored at −20 °C until further analysis.

### 2.4. Determination of Total Phenolic Compounds (TPC)

The total phenolic content (TPC) was determined by the Folin–Ciocalteu colorimetric assay using a method described by Arruda et al. [15]. Briefly, 100 µL of properly diluted extract was mixed with 100 µL of 50% (*v*/*v*) Folin–Ciocalteu reagent and 800 µL of 5% (*w*/*v*) sodium carbonate solution. Then, the reaction solution was stirred on a vortex mixer and kept at rest, protected from light, for 20 min at room temperature. The absorbance was measured at 760 nm against a blank on a spectrophotometer (Beckman, model DU600, Fullerton, CA, USA). Gallic acid was used as a standard, and the results were expressed as mg gallic acid equivalents per gram of dried pulp (mg GAE/g dw).

### 2.5. Trolox Equivalent Antioxidant Capacity (TEAC) Assay Using ABTS^•+^ radical

The Trolox equivalent antioxidant capacity (TEAC) of the araticum pulp extract was evaluated using the radical cation ABTS^•+^ according to the method described by Arruda et al. [15]. The radical cation ABTS^•+^ was generated by mixing 5 mL of a 7 mmol/L ABTS solution in water with 88 μL of a 145 mmol/L potassium persulfate solution. This mixture was allowed to stand overnight in the darkness at room temperature to ensure radical formation and stabilization. The ABTS^•+^ working solution was prepared by diluting the stock solution in ultrapure water to achieve an absorbance of 0.72 ± 0.02 at 734 nm. The reactions were conducted by mixing 200 µL of the appropriately diluted extract with 1 mL of ABTS^•+^ working solution for 6 min at room temperature. Subsequently, the remaining absorbance of ABTS^•+^ was measured at 734 nm against a blank in a spectrophotometer. Trolox, a compound recognized as an antioxidant reference, was used as a comparison standard, and the results were expressed as micromoles of Trolox equivalents per gram of dried pulp (µmol TE/g dw).

### 2.6. Oxygen Radical Absorbance Capacity (ORAC) Assay

The oxygen radical absorbance capacity (ORAC) assay was carried out based on a method previously described by Dávalos et al. [17]. Briefly, 20 μL of the appropriately diluted extract, 120 μL of the fluorescein in potassium phosphate buffer (0.378 μg/mL, pH 7.4), and 60 μL of AAPH in potassium phosphate buffer (108 mg/mL, pH 7.4) were added to each well of a 96-well dark microplate. For the blank, the extract was replaced with potassium phosphate buffer. Then, the microplate was incubated at 37 °C, and the fluorescence (excitation and emission wavelengths of 485 and 520 nm, respectively) was recorded every minute for 80 min on a NovoStar Microplate reader (New Brunswick Scientific Classic Series, model C76, Edison, NJ, USA). The final results were calculated using the difference between the areas under the fluorescence decay curves of extract and blank. Trolox was used as the reference antioxidant standard, and the results were expressed as micromoles of Trolox equivalents per gram of dried pulp (µmol TE/g dw).

### 2.7. Phytochemical Profile Analysis by UHPLC-ESI-QTOF-MS/MS

The profile of phytochemical compounds present in araticum pulp extract was achieved using an ultra-high-performance liquid chromatography (UHPLC) system (Agilent Technologies 1290 series Infinity System LC, Santa Clara, CA, USA) coupled to a Q-ToF iFunnel 6550 mass spectrometer with an electrospray ionization (ESI) interface in negative ionization mode under the following conditions: drying gas flow at 12 L/min; gas temperature at 290 °C; sheath gas temperature at 350 °C; VCap 3000 V; fragmentor voltage at 150 V; and OCT 1RF Vpp at 750 V. The separation of compounds was performed on a Poroshell 120 SB-Aq column (100 × 2.1 mm i.d., particle size 2.7 μm, Agilent Technologies, Santa Clara, CA, USA) using 0.1% formic acid in water (eluent A) and acetonitrile containing 0.1% formic acid (eluent B) as mobile phases. The elution gradient was performed as follows: 0–1 min, 5% B; 1–10 min, 5–18% B; 10–13 min, 18–70% B; 13–15 min, 70–100% B; 15–17 min, 100% B; and 17–20 min, 5% B. The flow rate was 0.45 mL/min, and the column temperature was kept at 40 °C. Mass spectra were acquired in profile mode, and the acquisition range was 100–1200 m/z [15]. Data were acquired and treated using Agilent MassHunter Qualitative Analysis B.07.00 software. For each signal obtained in the MS experiment, the molecular formula was proposed by the MassHunter Qualitative Analysis B.07.00 software and compared with previously reported phytochemicals in fruits, whose maximum error accepted for tentative identification was 8 ppm. The final identification of each compound was undertaken by manual interpretation of MS/MS pattern spectra, comparing the experimental fragmentation patterns with those available in databases, libraries, and scientific articles for phytochemicals.

### 2.8. Data Analysis

All experiments were carried out in triplicate, and all values were expressed as mean values ± standard deviation. Software from Microsoft Office Excel 2019 was used for the final data analysis.

## 3. Results and Discussion

The literature has been consistent in demonstrating that araticum pulp has a high content of phenolic compounds and potent antioxidant activity, as observed in the data presented in a recent review article published by our research group [6]. Various phytochemicals, particularly phenolic compounds, have been associated with these antioxidant effects. Despite recent efforts to identify these phytochemicals in araticum pulp, there are still a limited number of studies with this approach, and detailed characterization has not yet been achieved. Therefore, in the present study, we investigated the antioxidant potential of araticum pulp and conducted a comprehensive characterization of its phytochemical profile using UHPLC-ESI-QTOF-MS/MS.

### 3.1. Total Phenolic Content and Antioxidant Activities

Table 1 summarizes the results regarding the total phenolic content and antioxidant activities of the araticum pulp extract. The total phenolic content was estimated in the extract using the Folin–Ciocalteu method, which measures the reduction of the Folin–Ciocalteu reagent by phenolic compounds in an alkaline medium (pH close to 10), resulting in the formation of a blue complex that can be measured at 760 nm [18]. As shown in Table 1, the amount of extractable total phenolic compounds was 21.74 mg GAE/g dw (6.68 mg GAE/g fw). According to Vasco et al. [19], fruits can be classified into three categories based on their total phenolic content: low (<1 mg GAE/g), medium (1–5 mg GAE/g), and high (>5 mg GAE/g) for samples based on fresh weight (fw). Therefore, the araticum pulp can be considered a source of phenolic compounds since it exhibited a high content of these phytochemicals (6.68 mg GAE/g fw). The total phenolic content found in the present study was higher than that previously reported by Arruda et al. [8] (11.46 mg GAE/g dw), who also extracted phenolic compounds from araticum pulp using ultrasound-assisted extraction with a methanol–acetone–water (7:7:6, *v*/*v*/*v*) solvent system. The higher value of total phenolic content reported in our study can be explained by different factors, including the mode of applying acoustic energy and post-extraction steps. In the present study, an ultrasonic probe-based system was used in the extraction process, whereas Arruda et al. [8] performed ultrasound-assisted extraction by applying acoustic energy through an ultrasound bath. The mode of applying acoustic energy can profoundly affect the extraction yields. Ultrasonic probe-based systems are more efficient in extracting phenolic compounds from plant matrices because they allow for greater energy transfer to the extraction system due to the direct contact between the ultrasound probe and the sample, as well as the smaller volume of solvent in which the acoustic waves are dispersed compared to the ultrasonic bath [20]. Indeed, a recent study conducted by our group demonstrated that ultrasound-assisted extraction using a probe-based system (600 W) efficiently extracted phenolic compounds and antioxidants from araticum peel in a short processing time (5 min) [15]. Furthermore, in our study, we evaluated a crude extract, whereas Arruda et al. [8] performed a semi-purification step after extraction (liquid–liquid partitioning with diethyl ether–ethyl acetate (1:1, *v*/*v*)) that may have removed some interfering substances, particularly water-soluble non-phenolic reducing compounds. These non-phenolic substances (e.g., sugars, amino acids, proteins, vitamins, organic acids, inorganic ions, and metal complexes) may be present in higher quantities in crude extracts and could react with the Folin–Ciocalteu reagent, resulting in higher estimated values of total phenolic compounds in crude extracts compared to semi-purified or purified extracts [8,21].

Several other studies have estimated the total phenolic content of araticum pulp using the Folin–Ciocalteu method. The total phenolic content found in this study was lower, similar, or higher than those previously reported in other studies, as shown in Table 1. In addition to the aspects discussed above regarding the extraction method and extraction conditions, these discrepancies in the values of total phenolics among different studies can also be explained by other factors. The type and polarity of the extractor solvent are crucial for the extraction yield and composition profile of phenolic compounds from a plant matrix as they determine the partition selectivity of both phenolic compounds and potential interferents in the extraction system. Thus, the different extractor solvents used in the studies can, at least partially, explain the different values of total phenolic content found [31]. However, several studies used the same conditions to extract phenolic compounds from araticum pulp (successive extraction using 50% methanol and 70% acetone) and obtained discrepant results (total phenolic content values ranging from 2.22 to 10.08 mg GAE/g fw) [9,10,25,26,27,28,29]. Therefore, other factors such as geographical and environmental conditions of the fruit’s origin region (e.g., temperature, soil, incidence and intensity of light, harvest time, among others), physiological and genetic factors of the plant, and sample preparation and storage conditions may be associated with the observed variations in total phenolic values among studies [14,25]. In fact, the geographical and environmental conditions of the fruit’s origin region can strongly affect the phenolic compound content of araticum pulp. Ramos et al. [25] evaluated the content and profile of phenolic compounds in araticum fruits from different locations in the state of Minas Gerais and observed that the place of origin influenced both the profile and content of phenolic compounds (total phenolic content values ranging from 4.81 to 10.08 mg GAE/g fw) found in the fruit.

Several studies have demonstrated a strong and positive correlation between the content of phenolic compounds and the antioxidant activity of different plant matrices [16,32,33]. Arruda et al. [8] found significant correlations between the phenolic compounds (both total phenolic content and some individual phenolic compounds) and the antioxidant activity of araticum pulp. Phenolic compounds can exert their antioxidant activity through various mechanisms, including acting as reducing agents and hydrogen donors, chelating transition metals, scavenging and suppressing reactive species, inhibiting the expression and/or activity of enzymes involved in oxidative stress, and upregulating and/or protecting endogenous defense systems [7]. Therefore, the antioxidant activity of food matrices should be evaluated using more than one method to address different action mechanisms, thus obtaining more useful and consistent information regarding the antioxidant potential of the matrix in question [34]. Many antioxidant assays based on different action mechanisms have been proposed and employed to assess the antioxidant activity of plant matrices. Thus, to characterize the antioxidant activity of araticum pulp, the TEAC and ORAC assays were used, and the results are presented in Table 1.

The TEAC assay is based on the ability of antioxidants in the sample to reduce radical cation ABTS^•+^ through electron and/or hydrogen atom transfer. On the other hand, the ORAC assay is based on the ability of antioxidant compounds to inhibit peroxyl radicals through hydrogen atom transfer [22]. As seen in Table 1, araticum pulp showed a higher antioxidant activity value in the TEAC assay (218.11 µmol TE/g dw or 67.07 µmol TE/g fw), followed by the ORAC assay (172.90 µmol TE/g dw or 53.17 µmol TE/g fw). This indicates that the antioxidant compounds present in araticum pulp act efficiently through both electron and hydrogen atom transfer mechanisms. The antioxidant activity of araticum pulp obtained from both assays conducted in the present study was higher than the values previously reported by Arruda et al. [8] (115.30 and 140.07 μmol TE/g dw for TEAC and ORAC assays, respectively), who performed the extraction under similar conditions (ultrasound-assisted extraction using methanol–acetone–water (7:7:6, *v*/*v*/*v*) as the extractor solvent). The reasons for these discrepancies between the studies have been previously discussed when presenting the results of total phenolic content. Other previous studies have also evaluated the antioxidant activity of araticum pulp using the TEAC and ORAC assays, and the values are reported in Table 1. As discussed earlier when presenting the results of total phenolic content, variations in the antioxidant activity values found in different studies can be attributed to differences in fruit maturity, geographical location, climatic and environmental conditions of the fruit’s origin region, genetic and physiological factors of the plant, handling during and post-harvest, sample preparation and storage conditions, extraction method, and extraction conditions, among others [7,19].

### 3.2. Phytochemical Profile by UHPLC-ESI-QTOF-MS/MS

The identification/tentative annotation of the phytochemicals in the araticum pulp extract were achieved by UHPLC-ESI-QTOF-MS/MS. Non-targeted metabolite profiling and processing of UHPLC-ESI-QTOF-MS/MS data were performed using Agilent MassHunter Qualitative Analysis B.07.00 software. The characterization strategy was based on the exact mass (mass accuracy limit of 8 ppm), fragmentation patterns, and a comparison with the data available in the existing literature and databases for plant phytochemicals (e.g., MassBank (http://massbank.jp (accessed on 7 May 2023)), Food (https://foodb.ca (accessed on 13 May 2023)), METLIN Metabolite (http://metlin.scripps.edu (accessed on 20 May 2023)), and HMDB (https://hmdb.ca (accessed on 30 May 2023)). Fragmentation patterns (main MS/MS fragment ions) along with exact masses of the precursor ions in negative ionization mode (experimental *m*/*z* of deprotonated molecular ions ([M − H]^−^ or [M + COOH]^−^), molecular formula, error (ppm), retention time (min), and tentative identification for each phytochemical found in the araticum pulp extract are shown in Table 2. One hundred thirty-nine compounds belonging to different classes of phytochemicals were tentatively annotated and characterized based on their MS and MS/MS data in the araticum pulp, including four organic acids, seven jasmonates, four iridoid glycosides, forty-four flavonoids, thirty-eight non-flavonoid phenolic compounds, three alkaloids, eighteen annonaceous acetogenins, ten fatty acid derivatives, and eleven other compounds. Among these tentatively annotated compounds, 116 (about 83.45% of phytochemicals) were reported in the araticum pulp for the first time.

#### 3.2.1. Organic Acids

Four organic acids (compounds **1**–**4**) were tentatively identified in the araticum pulp, two of which were annotated for the first time: *n-*propylmalic acid and 2-furoic acid. On the other hand, malic and citric acids have already been widely reported in araticum pulp in previous studies [11,25,30,77]. Organic acids belong to an important class of organic compounds that contribute to the flavor of fruits and vegetables. Malic, citric, tartaric, succinic, and quinic acids are the main organic acids responsible for the flavor notes of most fruits [78]. According to Damiani et al. [30], malic and citric acids are the predominant organic acids present in araticum pulp (958.5 and 294.0 μg/g fw, respectively), suggesting that they are the main organic acids contributing to the fruit’s flavor. The high content of malic acid in araticum pulp reveals that this fruit belongs to the malic acid-dominant type of fruit. *n-*Propylmalic acid is a derivative of malic acid obtained through the addition of a propyl group to one of the carboxyl groups of malic acid. One of the few studies that reported the presence of malic acid in fruits was conducted by Cui et al. [79]. In this study, the authors evaluated the concentrations of different organic acids derived from malic acid during the ripening of Shushanggan apricot fruits. They observed a significant increase in 2-isopropylmalic acid, 2-propylmalic acid, and 3-isopropylmalic acid with a simultaneous reduction in malic acid throughout the fruit’s ripening, indicating that malic acid is being metabolized into these compounds. Therefore, the presence of *n-*propylmalic acid in the araticum pulp may be due to the metabolism of malic acid (the main organic acid present in araticum fruit) during the fruit ripening process. In addition, organic acids can play a critical role in plant adaptation to environmental stress. For example, the accumulation of certain organic acids when plants are subjected to drought stress can help with osmoregulation and maintain the balance of vesicle osmotic potential [80]. Zhang et al. [80] observed upregulation of various organic acids in licorice (*Glycyrrhiza uralensis* Fisch.) under severe drought stress, including 2-isopropylmalic acid, 3-isopropylmalic acid, and 2-propylmalic acid. The Cerrado, the biome where araticum trees have adapted, experiences long periods of drought throughout the year [4]. Thus, *n-*propylmalic acid production may be a response to the drought stress that the plant experiences in this environment. 2-Furoic acid has been found in fruits or fruit-based products as a product resulting from the degradation of ascorbic acid [81]. Although it was not detected in our study, ascorbic acid has been reported in araticum pulp in several studies. For example, Cardoso et al. [82] reported a quantity of 5.23 mg/100 g of ascorbic acid in araticum pulp. Therefore, the presence of this compound in the araticum pulp analyzed in the present study may be a result of the degradation of ascorbic acid. 2-Furoic acid, along with other furan derivatives, can contribute to impressions of fruity, caramel-like, sweet, nutty, meaty, and burnt odors in food products [83]. The present results demonstrate that malic acid, citric acid, and *n-*propylmalic acid can contribute to the slightly acidic taste of the araticum fruit, while 2-furoic acid may be responsible for the sweet and fruity odor notes. Additionally, the presence of *n-*propylmalic acid may be related to the plant’s defense mechanisms against the severe drought stress it experiences in the Cerrado environment.

#### 3.2.2. Jasmonates

Seven jasmonates (compounds **5**–**11**) were tentatively identified in the araticum pulp, all reported for the first time in the edible part of this fruit. Among the seven identified compounds, there were two isomers of dihydroxyjasmononic acid hexoside (compounds **5** and **6**), four isomers of tuberonic acid hexoside (compounds **7**–**10**), and (−)-11-hydroxy-9,10-dihydrojasmonic acid 11-β-D-glucoside (compound **11**). Jasmonates are oxylipin-type phytohormones derived from the oxidation of α-linolenic acid in the chloroplast membrane that occurs in different branches of the lipoxygenase pathway. These phytohormones are involved in signaling pathways related to plant growth, development, secondary metabolism, defense against biotic agents (e.g., insect attacks, pathogens, and herbivores), and tolerance to abiotic stresses (e.g., wounds, UV light, salt, drought, nutrient deficiency, and cold or heat) [84]. As mentioned in the Introduction section, the Brazilian Cerrado is a hostile environment that exposes its vegetation to a series of abiotic stresses (e.g., exposure to high temperatures and a water deficit for most of the year, increased incidence of UV radiation, frequent wildfires, and nutrient-poor soil) and biotic stresses (e.g., recurrent attacks by insects and pathogenic microorganisms) [4]. Thus, the production of jasmonates in araticum three may be one of several adaptations that this plant has undergone throughout its evolutionary process to resist the stressful conditions to which it is subjected in this biome and, consequently, to adapt and survive in it. Therefore, the presence of jasmonates in the araticum fruit, as observed here, may be of fundamental importance for the development and tolerance of the fruits to the biotic and abiotic stressful factors of the Brazilian Cerrado environment.

#### 3.2.3. Iridoids

Four iridoids (compounds **12**–**15**) have been tentatively identified in araticum pulp, and all of them have been reported for the first time in this part of the fruit. All identified iridoids were bound to at least one sugar unit, with two belonging to the class of iridoid glycosides (ajugol and aucubin) and the other two to secoiridoid glycosides (two isomers of aldosecologanin). Iridoids are a class of phytochemicals belonging to the monoterpenoids with a general cyclopentopyran form and a molecular structure related to iridodial. The C_1_–OH group of iridoids is often unstable and readily reacts with sugars to form glycosides, which may explain the presence of only glycosylated iridoids in the araticum pulp. In plants, iridoids serve defensive functions against viruses and microorganisms and quickly repair damaged areas. Furthermore, these phytochemicals protect plants from insect and herbivore attacks due to their discouraging bitter taste [85,86]. Therefore, the presence of these compounds in araticum fruit may be related to their role in protecting the fruit against viruses, microorganisms, insects, and herbivore attacks, particularly during its developmental stages. Moreover, recent studies have demonstrated that these phytochemicals exhibit pharmacological effects, including anti-inflammatory, hypolipidemic, hypoglycemic, hepatoprotective, neuroprotective, and anticancer activities, among others [85]. Therefore, consuming araticum fruit may promote the health benefits associated with iridoid glycosides. However, further studies on the characterization and quantification of these compounds in the fruit are needed to better understand whether the consumed amounts would be sufficient to promote health and well-being in humans.

#### 3.2.4. Phenolic Compounds

Phenolic compounds are the most extensively documented group of secondary metabolites in the araticum pulp to date [8,9,11,14,25]. These compounds are characterized by having at least one aromatic ring with one or more hydroxyl substituent groups. In addition, they can be linked or not to other substances (e.g., carbohydrates, amines, lipids, organic acids, and cell wall components) [4]. As shown in Table 2, 82 phenolic compounds (compounds **16**–**97**) belonging to different classes have been tentatively identified in the araticum pulp, representing approximately 59% of all the identified phytochemicals. Quantitatively, flavonoids (compounds **16**–**59**, totaling 44 different compounds) were the main class of phenolic compounds identified in the araticum pulp (approximately 54% of the total identified phenolic compounds), while 38 non-flavonoid phenolic compounds (compounds **60**–**97**) were also tentatively annotated. Some studies have shown upregulation of gene expression related to flavonoid biosynthesis in plants under excess light and drought stress [80,87]. This may explain the accumulation of flavonoids in the araticum fruit since the plant undergoes both stresses in the Cerrado environment.

Most of the phenolic compounds present in the araticum pulp (43 compounds: compounds **28**, **37**, **39**, **40**, **43**–**47**, **50**–**57**, **60**–**77**, **79**–**82**, **85**, **87**, **90**, and **92**) were found to be linked to one or more sugar residues, indicating that the phenolic compounds in this fruit are predominantly in glycosylated form. Phenolic compounds, as well as other secondary metabolites, play a key role in the early stages of plant defense against different biotic and abiotic agents. However, these compounds can also be toxic to the plant itself. Thus, plants have developed strategies throughout their evolutionary process to overcome this drawback, among which the conjugation of these toxic defense compounds with different organic molecules, including carbohydrates, stands out. Plant glycosyltransferase enzymes act on the aglycone forms of phenolic compounds, binding them to sugar units and consequently generating non-toxic or less toxic agents that are often sequestered in a storage compartment, such as cellular vacuoles. When the plant undergoes an attack, the detoxified phenolic compounds are activated by the action of glycosylhydrolase enzymes released by the plant itself or by the invading organism, allowing them to act in plant defense processes. Additionally, glycosylation of phenolic compounds increases their solubility and stability within the cellular environment, improving their biodistribution and metabolism. As a result, phenolic compounds accumulate in plant cells, particularly in their glycosylated forms [88,89]. These aspects could explain why phenolic compounds are predominantly stored in the tissues of araticum fruit in glycosylated form, as observed here.

Forty-four flavonoids (compounds **16**–**59**) were tentatively annotated in araticum pulp, including twenty-five flavanols (compounds **16**–**20**, **22**–**27**, **29**–**36**, **38**, **40**, **41**, **48**, **49**, and **58**), thirteen flavonols (compounds **39**, **40**, **43**, **44**, **46**, and **50**–**57**), two dihydroflavonols (compounds **21** and **28**), two flavanones (compounds **37** and **45**), one flavone (compound **59**), and one dihydrochalcone (compound **47**). Flavanols and flavonols were the main classes of flavonoids found in araticum pulp, accounting for over 86% of the identified flavonoids. In fact, Arruda et al. [8] also found that flavanols were the main class of flavonoids present in araticum pulp. The flavanols found in araticum pulp were primarily composed of catechin and epicatechin oligomers (known as procyanidins), while flavonols were predominantly composed of quercetin and kaempferol glycosides. Some of the identified flavonoids have been previously reported in araticum pulp in other studies, including catechin [8,9,11], epicatechin [8,14,25,90], rutin [8,9,14], quercetin-3-*O*-β-D-glucoside [14,25], hesperidin [25], procyanidin A dimer [25], procyanidin B trimer [25], procyanidin B2 dimer [14], kaempferol-3-*O*-rutinoside [14], and kaempferol-3-*O*-β-D-glucoside [14]. Meanwhile, thirty-four flavonoids are being reported for the first time in the present study, namely four procyanidin dimer isomers, eight procyanidin trimer isomers, seven procyanidin tetramer isomers, two quercetin-3-*O*-pentosylhexoside isomers, two kaempferol hexoside isomers, kaempferol-3-*O*-hexosylpentoside, dihydrokaempferol hexoside, kaempferol deoxyhexosylhexoside, dihydroquercetin hexoside, flavanomarein, phloretin-*C*-diglycoside, quercetin pentoside, isorhamnetin-3-*O*-rutinoside, isorhamnetin hexoside, (epi)catechin-ethyl trimer, and luteolin.

In addition to flavonoids, thirty-eight non-flavonoid phenolic compounds (compounds **60**–**97**) were tentatively identified in araticum pulp, including twenty-eight phenolic acids (compounds **60**, **62**, **64**, **66**–**74**, **76**–**81**, **83**, **84**, **86**, **89**, **91**–**95**, and **97**), three lignans (compounds **87**, **88**, and **90**), and seven other phenolic compounds (five methoxyphenols (compounds **63**, **75**, **82**, **85**, and **96**) and two tyrosols (compounds **61** and **65**)). Phenolic acids were the predominant class among the non-flavonoid phenolic compounds present in araticum pulp, accounting for approximately 74% of all identified non-flavonoid phenolic compounds. Additionally, hydroxycinnamic acids (18 compounds: compounds **64**, **68**, **69**, **71**, **72**, **76**–**78**, **80**, **81**, **83**, **84**, **86**, **89**, **91**–**93**, and **95**) were the main phenolic acids found in araticum pulp, followed by hydroxybenzoic acids (eight compounds: compounds **60**, **62**, **66**, **67**, **73**, **74**, **79**, and **97**), hydroxyphenylpropanoic acids (compound **70**), and other phenolic acids (compound **94**). Arruda et al. [8] also reported a lower content of hydroxybenzoic acids in araticum pulp compared to hydroxycinnamic acids. These data corroborate the literature, which has reported that hydroxybenzoic acids are generally found in low concentrations in food plants [21]. Hydroxycinnamic acids and hydroxybenzoic acids were the major subclasses of non-flavonoid phenolic compounds in araticum pulp, with hydroxycinnamic acids primarily composed of derivatives of caffeic acid, ferulic acid, and *p*-coumaric acid, while derivatives of vanillic acid and dihydroxybenzoic acids were the predominant compounds in hydroxybenzoic acids. Some of these non-flavonoid phenolic compounds have been previously identified in araticum pulp, including chlorogenic acid [8,9,25], caffeic acid [8,9], ferulic acid [8,9,11,14,25], dihydrocoumaroyl hexoside [25], *p*-coumaric acid methyl ester [25], *p*-coumaric acid hexoside [25], ferulic acid hexoside [25], syringic acid hexoside [25], and hydroxytyrosol hexoside [25]. On the other hand, twenty-nine non-flavonoid phenolic compounds were identified here for the first time, namely protocatechuic acid hexoside, hydroxybenzoic acid hexoside, leonuriside A, two caffeoylsucrose isomers, hydroxytyrosol hexosylpentoside, three vanillic acid hexoside isomers, two dihydroxybenzoic acid pentoside isomers, three caffeic acid hexoside isomers, two caffeoyltyramine isomers, coniferin, methylsyringin, caffeoylshikimic acid, phloroacetophenone 6′-[xylosyl-(1→6)-glucoside], pinoresinol hexoside, pinoresinol, syringaresinol-*O*-β-D-glucopyranoside, caffeic acid ethyl ester, lavandulifolioside, *N-*feruloyltyramine, 4-hydroxyphenyl-hexanoic acid, verimol H, and *p-*decycloxybenzoic acid.

Phenolic compounds are widely recognized for their potent antioxidant properties. As discussed in Section 3.1, araticum pulp exhibited high antioxidant activity in TEAC and ORAC assays. The antioxidant activity of a plant matrix strongly depends on the content and profile of phenolic compounds present in it. Structural chemical features of phenolic compounds, such as the number of aromatic rings and hydroxyl groups, the degree of hydrogen atom substitution by other functional groups, the degree of glycosylation, and the specific positioning of these groups in the molecule, can profoundly affect the antioxidant potential of a food. Additionally, different classes of phenolic compounds can synergistically interact with each other and with other components of the matrix, enhancing the overall antioxidant activity [15]. Therefore, the presence of a large and diverse number of phenolic compounds in araticum pulp may explain its high antioxidant activity. Apart from their antioxidant potential, the structural characteristics of phenolic compounds have been associated with a broad spectrum of bioactivities, including (but not limited to) anti-inflammatory, anticancer, antidiabetic, antidyslipidemic, anti-obesity, neuroprotective, hepatoprotective, and cardioprotective effects (for more details on the biological activities of phenolic compounds and their underlying mechanisms of action, please refer to the review conducted by Rana et al. [91]). Therefore, consuming araticum fruit can contribute to the intake of important bioactive phenolic compounds and, consequently, promote beneficial effects on human health and well-being. However, further in vivo studies should be conducted with araticum pulp to validate these effects.

#### 3.2.5. Alkaloids

Three alkaloids (compounds **98**–**100**) have been tentatively identified in araticum pulp, two of which belong to the class of heterocyclic alkaloids (bakankoside e isoboldine), and damascenine is classified as a non-heterocyclic alkaloid. Alkaloids are nitrogen-containing compounds that occur naturally in plants and serve as a defense mechanism to protect them from predators (e.g., herbivorous insects and vertebrates), pathogenic bacteria and fungi, and plant parasites [92]. Among the identified alkaloids, damascenine and bakankoside were reported for the first time in araticum pulp. On the other hand, isoboldine had been previously reported in this part of the fruit by Ramos et al. [90]. Like other phytochemicals described above, the presence of these alkaloids in araticum fruit may be associated with their role in regulating the fruit’s defense system against biotic and abiotic agents. Additionally, several in vivo studies and clinical investigations have demonstrated that alkaloids have various pharmacological effects, including anticancer, antiviral, anti-inflammatory, antimicrobial, antioxidant, antidiabetic, antihypertensive, antidiarrheal, and antimalarial activities. Due to their multiple biological activities, various medications derived from natural alkaloids are available on the market (for more details on the therapeutic uses of some alkaloids, refer to the reviews by Debnath et al. [93] and Bhambhani et al. [92]). Therefore, therapeutic effects could potentially be achieved through the consumption of araticum fruit. However, further in-depth studies are required regarding the characterization and quantification of alkaloids in the pulp of this fruit to better understand their potential relationship with health benefits and the quantities present in a fruit serving.

#### 3.2.6. Annonaceous Acetogenins

Eighteen different annonaceous acetogenins (compounds **101**–**118**) were tentatively annotated for the first time in araticum pulp. Annonaceous acetogenins are a series of natural polyketides found almost exclusively in plants of the Annonaceae family. These phytochemicals constitute a unique class of C_35_ or C_37_ secondary metabolites derived from long-chain fatty acids, predominantly lacceroic acid (C_32_) or ghedoic acid (C_34_), which are combined with a 2-propanol unit at C_2_, forming a methyl-substituted α,β-unsaturated γ-lactone ring (sometimes rearranged to a ketolactone) [94,95]. Nearly 600 annonaceous acetogenins have already been identified from 51 species in 13 genera [72]. Many annonaceous acetogenins are isomers, thus having the same molecular formula but differing from each other only by the location of the tetrahydrofuran rings and hydroxyl groups along the hydrocarbon chain. This structural diversity greatly hinders the differentiation between the isomeric forms during structural characterization. In the present study, we obtained mass spectra and fragmentation pathways for all the phytochemicals using an electrospray ionization source (ESI-MS/MS) operating in negative mode. Generally, the fragmentation pathways of annonaceous acetogenins are obtained in positive mode, with a very limited number of available scientific studies and databases reporting fragmentation pathways in negative mode, making it challenging to fully assign the tentatively identified acetogenins in araticum pulp. Thus, the identification of these 18 compounds as belonging to the class of annonaceous acetogenins was possible due to structural chemical features obtained from both the exact mass of the precursor ion ([M − H]^−^) and some characteristic fragments of acetogenins obtained from the precursor ion. Analysis of the fragmentation pathways and characteristic fragments of annonaceous acetogenins present in araticum pulp obtained in the negative mode was conducted according to Allegrand et al. [73], who described the fragmentation pathways of acetogenins (particularly annonacin) during ESI-MS/MS experiments in the negative mode. According to this study, the most characteristic fragments of annonaceous acetogenins are a fragment ion at *m*/*z* 127.04 [C_6_H_7_O_3_]^−^ corresponding to the tetrahydrofuran ring, losses of water and/or CO_2_, and other typical fragments. All the identified annonaceous acetogenins here had 35 or 37 carbon atoms and presented one or more of these characteristic fragments of this compound class. All the tentatively annotated annonaceous acetogenins in araticum pulp exhibited the fragment ion at *m*/*z* 127.04 [C_6_H_7_O_3_]^−^ corresponding to the tetrahydrofuran ring, indicating that all of them belonged to the tetrahydrofuran acetogenin class. Furthermore, several fragment ions formed by successive losses of water and/or CO_2_ molecules from the precursor molecular ion ([M − H]^−^) were also found in all tentatively annotated anonaceous acetogenins in the araticum pulp. Other typical fragment ions of anonaceous acetogenins, such as fragment ions produced by cleavage in the alpha of the first hydroxyl group starting from the methyl extremity (*m*/*z* 197.19 [C_13_H_25_O]^−^) and fragment ions resulting from the loss of the terminal γ-lactone ring (loss of 112 u corresponding to fragment ion [M-H-C_6_H_8_O_2_]^−^), were also reported in several of the tentatively annotated anonaceous acetogenins in the araticum pulp. Detailed information regarding the specific fragmentation pathways of the tentatively identified anonaceous acetogenins in the araticum pulp is presented in Table 3.

The identification of annonaceous acetogenins in the araticum pulp was achieved to some degree, while precise identification and quantification of the individual compounds remain to be performed. In plants, these secondary metabolites derived from the oxylipin pathway are involved in the plant’s defense mechanisms against pests and pathogens [94]. Therefore, the presence of annonaceous acetogenins in the araticum fruit may be particularly associated with its action as a protective barrier against insect and pathogen attacks. In fact, several studies have demonstrated the antimicrobial, antiparasitic, and pesticidal potential of isolated annonaceous acetogenins and/or acetogenin-rich extracts [95,96]. Furthermore, these compounds have emerged as potent anticancer, cytotoxic, and immunosuppressive agents [96]. Recent studies have highlighted the selective cytotoxic effect of annonaceous acetogenins against various human tumor cell lines, demonstrating their potential for controlling different types of cancer [6]. Thus, araticum pulp may be a promising food source to prevent the onset and/or slow the progression of cancer. However, further studies are needed to characterize and quantify the annonaceous acetogenins present in araticum pulp, as well as interventional studies in animals and humans to verify the actual effects of consuming this fruit on the prevention and/or control of tumoral processes.

#### 3.2.7. Fatty Acid Derivatives

Due to the highly hydrophobic nature of fatty acids, it was not expected to find these compounds in the analyzed extract. However, ten fatty acid-derived compounds (compounds **119**–**128**) were tentatively annotated in this araticum pulp extract, all for the first time. The presence of these compounds is attributed to certain polar groups in their structures (e.g., sugar moieties, hydroxyl groups, and hydroperoxyl groups), which make them more soluble in the extractor solvent used. Fatty acids play different roles in plants, including structural functions as constituents of phospholipids that compose the cell membrane, serving as a source of energy reserves in cells, and acting as precursors to bioactive molecules involved in cellular signaling and response to environmental stresses and pathogen attacks. Thus, their presence and metabolism are crucial for the development and adaptation of the plant to the environment [97]. Most of the fatty acid derivatives identified in araticum pulp were found to be linked to carbohydrates (70% of fatty acid derivatives). Similar to glycosphingolipids, a class of glycosylated lipids, glycosylated fatty acid derivatives can act as mediators of plant growth, reproduction, and defense [98]. For example, the production of 5-hydroxyhexanoic acid 3-*O*-β-D-glucoside was upregulated in powdery mildew-resistant gerbera varieties (*Gerbera hybrida*), suggesting that this compound may play a role in the plant’s resistance to powdery mildew [89]. The significance and specific biological function of glycosylated fatty acid derivatives may vary depending on the context and organism in which they are found. Therefore, future research is necessary to determine the specific functions and potential biological activities associated with these glycosylated fatty acid derivatives found specifically in araticum pulp.

In addition to glycosylated fatty acid derivatives, two oxylipins were also found in the araticum pulp extract: 9,12,13-trihydroxy-octadecadienoic acid and 11-hydroperoxy-octadecatrienoic acid. Oxylipins are a family of oxygenated products derived from fatty acids and generated through autoxidation or enzymatic oxidation of polyunsaturated fatty acids. These compounds also play important roles in plants, acting as signaling molecules between plants, defending the plant against pathogens, and responding to stressful environmental conditions [65]. In fact, Göbel et al. [99] observed an accumulation of oxylipins, particularly trihydroxy oxylipins, in *Phytophthora infestans* elicitor-treated cultured potato cells, supporting the idea that these compounds may be involved in plant defense reactions. Thus, glycosylated fatty acid derivatives and oxylipins found in araticum pulp may be important phytochemicals involved in plant development and defense in the Cerrado environment. On the other hand, there are no reports in the literature regarding the role of 6E-octene-2,4-diynoic acid in plants, thus necessitating further studies to determine its specific function.

#### 3.2.8. Other Compounds

In addition to the aforementioned classes, 11 other compounds (compounds **129**–**139**) belonging to different classes of phytochemicals were tentatively identified in araticum pulp.

L-Arginine, a conditionally essential amino acid, was identified for the first time in araticum pulp in its free form. L-Arginine was the main free amino acid found in cherimoya (*Annona cherimola* Mill.) pulp, accounting for approximately 72% of all quantified amino acids, while the other free amino acids were found in significantly lower amounts [100]. The identification of only L-arginine in the analyzed extract may indicate that it is also the main free amino acid present in araticum pulp. L-Arginine plays significant metabolic and regulatory roles in humans and has been shown to reverse endothelial dysfunction, improve wound healing, prevent tumor development, and enhance immune, reproductive, renal, cardiovascular, digestive, and pulmonary functions [100].

Two charged acidic sugars were also found in the analyzed araticum pulp extract: gluconic acid and glucuronic/galacturonic acid. Several neutral sugars were identified and quantified in araticum pulp by Arruda et al. [101], but charged sugars were not analyzed in this study. Glucuronic/galacturonic acid was identified here for the first time, while gluconic acid was previously reported in araticum pulp in a study conducted by Ramos et al. [25]. Gluconic acid is a derivative of glucose oxidation, while glucuronic and galacturonic acids are derived from the hydrolysis of polysaccharides such as pectin [102]. These acidic sugars can be formed during fruit ripening and contribute to the sweet and slightly acidic taste of the araticum fruit.

A derivative of vitamin B5 (pantothenic acid hexoside) was tentatively identified in araticum pulp for the first time. Pantothenic acid is an essential nutrient with significant importance for the human body. It is an important precursor in the biosynthesis of coenzyme A, which plays a crucial role in promoting the growth of organisms [103]. Glycosylated forms of pantothenic acid have been identified in nature. Glycosylated pantothenic acid is generally formed from pantothenic acid and various glycosyl donors through the action of glycosidase enzymes (e.g., β-glucosidases) [104]. The presence of pantothenic acid in araticum pulp may suggest it is a potential source of this nutrient. However, further studies need to be conducted to identify and quantify pantothenic acid derivatives in araticum pulp to determine their actual contribution to the recommended daily intake of this nutrient.

Abscisic acid and its metabolite, dehydrophaseic acid hexoside, were tentatively identified in araticum pulp for the first time. Abscisic acid is a phytohormone related to the plant’s response to abiotic stresses, particularly drought. It regulates stomatal closure to reduce water loss, initiates gene transcription for additional water conservation measures, and stimulates the biosynthesis of flavonoids to prevent and counteract oxidative stress triggered by desiccation and high light irradiation [76,105]. The presence of dehydrophaseic acid hexoside may be related to the inactivation of abscisic acid, which can occur by oxidative conversion to phaseic acid or dehydrophaseic acid or by conjugation with glucose [106]. The accumulation of glycosylated hormonal metabolites during fruit development occurs due to the discontinuous need for the triggering hormonal molecules (aglycone forms) after fruit cell expansion [107]. Roseoside was another abscisic acid-related compound identified for the first time in araticum pulp. This megastigmane glycoside has been found in different plant species. The biological relevance of megastigmane accumulation in plants is not yet fully understood, but it may be related to defense against herbivores [108]. However, recent studies have demonstrated that roseoside has various biological properties of interest, including anticarcinogenic, antihypertensive, and antiallergic effects [109]. Therefore, the presence of these abscisic acid-related compounds in the araticum fruit may be related to the plant’s defense against the stressful factors in the Cerrado environment.

Another four compounds were tentatively identified for the first time in araticum pulp, namely benzyl-pentosylhexoside, methylbenzoic acid, 1-hexanol arabinosylglucoside, and (1S,2S,4R,8S)-*p-*menthane-1,2,8,9-tetrol-2-glucoside. These compounds have been identified in other plants and appear to be related to their aroma. For example, 1-hexanol has been associated with a fresh aroma [110], while benzyl alcohol has a slightly pungent sweet taste and a pleasant fruity odor [111]. However, these compounds may also have specific functions in plants, such as antioxidant and antimicrobial activity, which are not yet well understood. Therefore, further studies are needed to determine the specific functions of these compounds in plants as well as their potential effects on human health and well-being.

## 4. Conclusions

Araticum is one of the most important fruit species in the Brazilian Cerrado, representing a significant source of income and nutrients for the local population. However, the literature data on the phytochemicals of the fruit, particularly the edible part, are scarce. Thus, we conducted a comprehensive characterization of the phytochemical profile present in the araticum pulp using ultra-high-performance liquid chromatography coupled to a quadrupole time-of-flight mass spectrometer (UHPLC-ESI-QTOF-MS/MS). One hundred and thirty-nine secondary metabolites were tentatively identified, including organic acids, jasmonates, iridoids, phenolic compounds, alkaloids, anonaceous acetogenins, fatty acid derivatives, and other compounds. Most of these compounds are known to play key roles in the plant’s defense mechanisms against biotic and abiotic stress factors in the Cerrado environment. Among the tentatively annotated compounds, 116 were reported in the araticum pulp for the first time. Phenolic compounds were the major class of phytochemicals in the araticum pulp, representing more than half of all tentatively annotated compounds. Due to the large number of existing isomers, the identification of phenolic compounds and annonaceous acetogenins in the araticum pulp was achieved to some degree, while precise identification and quantification of the individual compounds remain to be performed. In addition to their important roles in the plant, a significant number of compounds annotated in the araticum pulp are known to exhibit various biological activities that can promote beneficial effects on human health and well-being. Therefore, antioxidant assays were performed to better understand the functional potential of this fruit. The strong antioxidant activity (TEAC and ORAC assays) of the fruit pulp may be particularly related to the presence of phenolic compounds. However, other classes of phytochemicals may also act individually or synergistically with each other and with the phenolic compounds, contributing at least in part to the fruit’s antioxidant activity. The data reported here provide new scientific insights into the phytochemical profile and biological effects (particularly antioxidant activity) of araticum pulp, demonstrating that this fruit can be a potential source of health-promoting compounds for the development of functional applications in the food, nutraceutical, and pharmaceutical industries. However, further studies on the bioavailability and in vivo assays (particularly animal studies and clinical trials) should be conducted with the fruit to better understand the stability and absorption of these bioactive compounds throughout the gastrointestinal tract, as well as their actual contribution to human health and well-being. Furthermore, there is a need to conduct future studies with plant populations from other cities/regions to obtain a more comprehensive phytochemical profile, better understand their genetic and geographic diversity, and generate a chemical fingerprint of this fruit.

## Figures and Tables

**Table 1:** Total phenolic content and antioxidant activity by TEAC and ORAC methods found in araticum pulp in our study and previous studies.

TPC (mg GAE/g)		TEAC (µmol TE/g)		ORAC (µmol TE/g)		Refs.
dw	fw	dw	fw	dw	fw	
21.74 ± 0.30	6.68 ± 0.09	218.11 ± 5.54	67.07 ± 1.70	172.90 ± 6.17	53.17 ± 1.90	Our study
11.46	-	115.30	-	140.07	-	[8]
46.70	-	683.65	-	546.26	-	[7]
45.58	-	184.81	-	-	-	[11]
26.20	-	231.79	-	337.25	-	[22]
15.89	-	94.66	-	-	-	[14]
12.45	-	-	-	-	-	[23]
5.80–10.95	-	-	-	-	-	[24]
-	4.81–10.08	-	23.16–93.76	-	-	[25]
-	7.39	-	131.58	-	-	[26]
-	7.28	-	132.16	-	-	[27]
-	4.34	-	-	-	-	[28]
-	2.90	-	-	-	-	[9]
-	2.58	-	-	-	-	[10]
-	2.22	-	-	-	-	[29]
-	2.11–2.61	-	-	-	-	[30]

dw: dried weight; fw: fresh weight; GAE: gallic acid equivalents; ORAC: oxygen radical absorbance capacity; TE: Trolox equivalents; TEAC: Trolox equivalent antioxidant capacity; TPC: total phenolic content.

**Table 2:** Identified or tentatively annotated phytochemicals found in the araticum pulp extract by UHPLC-ESI-QTOF-MS/MS under negative ion mode.

No.	Identified/Tentatively Annotated Compound	r.t. (min)	Experimental Mass (*m*/*z*)	Calculated Mass	MS/MS Fragment Ions (m/z)	Molecular Formula	Error (ppm)	Refs.
	*Organic acids and derivatives*							
1	Malic acid	0.67	133.0145 [M − H]^−^	134.0217	133, 115	C_4_H_6_O_5_	1.67	[35]
2	Citric acid	0.73	191.0205 [M − H]^−^	192.0278	191, 129, 111	C_6_H_8_O_7_	3.54	[36]
3	2-Furoic acid *	0.75	111.0088 [M − H]^−^	112.0162	111, 109, 106	C_5_H_4_O_3_	−1.39	[37]
4	*n-*Propylmalic acid *	1.49	175.0622 [M − H]^−^	176.0695	131, 115, 113	C_7_H_12_O_5_	−5.86	[15]
	*Jasmonates and derivatives*							
5	Dihydroxyjasmononic acid hexoside isomer 1 *	2.74	403.1618 [M − H]^−^	404.1701	223, 161	C_18_H_28_O_10_	−4.46	[38]
6	Dihydroxyjasmononic acid hexoside isomer 2 *	2.92	403.1612 [M − H]^−^	404.1685	241, 223, 179, 149	C_18_H_28_O_10_	−0.71	[38]
7	Tuberonic acid hexoside isomer 1 *	3.65	387.1671 [M − H]^−^	388.1745	387, 163, 119	C_18_H_28_O_9_	−3.11	[39]
8	Tuberonic acid hexoside isomer 2 *	4.09	387.1664 [M − H]^−^	388.1738	387, 163, 119	C_18_H_28_O_9_	−1.21	[39]
9	Tuberonic acid hexoside isomer 3 *	4.48	387.1654 [M − H]^−^	388.1727	387, 207, 163, 119	C_18_H_28_O_9_	1.63	[39]
10	Tuberonic acid hexoside isomer 4 *	4.65	387.1658 [M − H]^−^	388.1731	387, 207, 163, 119	C_18_H_28_O_9_	0.54	[39]
11	(−)-11-hydroxy-9,10-dihydrojasmonic acid 11-β-D-glucoside *	5.30	389.1823 [M − H]^−^	390.1895	389, 227, 133, 101	C_18_H_30_O_9_	−1.36	[40]
	*Iridoids*							
12	Ajugol *	2.83	393.1399 [M + COOH]^−^	348.1417	161, 141, 135, 119	C_15_H_24_O_9_	0.89	[41]
13	Aucubin *	3.05	391.1253 [M + COOH]^−^	346.1272	183, 168, 151, 123	C_15_H_22_O_9_	−2.35	[41]
14	Aldosecologanin isomer 1 *	11.02	757.2612 [M − H]^−^	758.2633	595	C_34_H_46_O_19_	−7.53	[42]
15	Aldosecologanin isomer 2 *	11.41	757.2616 [M − H]^−^	758.2633	595	C_34_H_46_O_19_	−8.06	[42]
	*Flavonoids and derivatives*							
16	Procyanidin A dimer	2.61	575.1195 [M − H]^−^	576.1267	449, 423, 407, 289, 125	C_30_H_24_O_12_	0.18	[43]
17	Catechin	3.39	289.0726 [M − H]^−^	290.0799	289, 245, 151, 137, 123, 109	C_15_H_14_O_6_	−2.97	[44]
18	Procyanidin B dimer isomer 1 *	3.52	577.1353 [M − H]^−^	578.1426	577, 451, 425, 407, 289, 161, 125	C_30_H_26_O_12_	−0.25	[45]
19	Procyanidin B trimer isomer 1	4.24	865.1998 [M − H]^−^	866.2069	865, 713, 695, 577, 543, 451, 425, 287, 125	C_45_H_38_O_18_	−0.84	[45]
20	Procyanidin B dimer isomer 2	4.44	577.1354 [M − H]^−^	578.1428	577, 451, 425, 407, 289, 161, 125	C_30_H_26_O_12_	−0.59	[45]
21	Dihydrokaempferol hexoside *	4.59	449.1091 [M − H]^−^	450.1165	449, 287, 269, 259, 179, 151	C_21_H_22_O_11_	−0.53	[46]
22	Epicatechin	4.70	289.0721 [M − H]^−^	290.0794	289, 245, 221, 205, 151, 137, 125, 123, 109	C_15_H_14_O_6_	−2.30	[44]
23	Procyanidin B dimer isomer 3 *	4.78	577.1364 [M − H]^−^	578.1438	577, 451, 425, 407, 289, 161, 125	C_30_H_26_O_12_	−2.29	[45]
24	Procyanidin B trimer isomer 2 *	4.90	865.1976 [M − H]^−^	866.2050	865, 713, 695, 577, 543, 451, 425, 287, 125	C_45_H_38_O_18_	0.93	[45]
25	Procyanidin B tetramer isomer 1 *	5.08	1153.2637 [M − H]^−^	1154.2709	1027, 865, 695, 577, 575, 533, 451, 449, 413, 287, 125	C_60_H_50_O_24_	−1.51	[45]
26	Procyanidin B trimer isomer 3 *	5.30	865.1993 [M − H]^−^	866.2067	865, 713, 695, 577, 543, 451, 425, 287, 125	C_45_H_38_O_18_	−1.04	[45]
27	Procyanidin B trimer isomer 4 *	5.56	865.1998 [M − H]^−^	866.2070	865, 713, 695, 577, 543, 451, 425, 287, 125	C_45_H_38_O_18_	−0.71	[45]
28	Dihydroquercetin hexoside *	5.99	465.1045 [M − H]^−^	466.1118	285, 151	C_21_H_22_O_12_	−1.39	[47]
29	Procyanidin B tetramer isomer 2 *	6.04	1153.2599 [M − H]^−^	1154.2672	1027, 1001, 983, 863, 533, 407, 289, 297	C_60_H_50_O_24_	1.77	[45]
30	Procyanidin B dimer isomer 4 *	6.19	577.1350 [M − H]^−^	578.1423	577, 451, 425, 407, 289, 161, 125	C_30_H_26_O_12_	0.15	[45]
31	Procyanidin B trimer isomer 5 *	6.30	865.1967 [M − H]^−^	866.2040	865, 713, 695, 577, 543, 451, 425, 287, 125	C_45_H_38_O_18_	2.14	[45]
32	Procyanidin B tetramer isomer 3 *	6.77	1153.2605 [M − H]^−^	1154.2675	1027, 1001, 983, 865, 863, 739, 695, 577, 575, 451, 449, 423, 413, 407, 289, 287, 125	C_60_H_50_O_24_	1.44	[45]
33	Procyanidin B trimer isomer 6 *	6.92	865.1978 [M − H]^−^	866.2049	865, 713, 695, 577, 543, 451, 425, 287, 125	C_45_H_38_O_18_	−1.03	[45]
34	Procyanidin B tetramer isomer 4 *	7.21	1153.2611 [M − H]^−^	1154.2683	1027, 1001, 865, 863, 575, 451, 459, 423, 413, 405, 289, 287	C_60_H_50_O_24_	0.75	[45]
35	Procyanidin B trimer isomer 7 *	7.60	865.1987 [M − H]^−^	866.2060	865, 713, 695, 577, 543, 451, 425, 287, 125	C_45_H_38_O_18_	0.27	[45]
36	Procyanidin B tetramer isomer 5 *	7.64	1153.2618 [M − H]^−^	1154.2685	1027, 983, 865, 863, 739, 695, 577, 575, 451, 449, 407, 289, 287, 125	C_60_H_50_O_24_	0.61	[45]
37	Flavanomarein *	7.74	449.1088 [M − H]^−^	450.1160	449, 269, 179, 151, 135	C_21_H_22_O_11_	0.54	[48]
38	Procyanidin B tetramer isomer 6 *	8.03	1153.2621 [M − H]^−^	1154.2696	1001, 983, 863, 695, 577, 575, 451, 449, 413, 407, 289, 287, 125	C_60_H_50_O_24_	−0.36	[45]
39	Quercetin-3-*O*-pentosylhexoside isomer 1 *	8.20	595.1307 [M − H]^−^	596.1381	595, 301, 300, 271, 179, 151	C_26_H_28_O_16_	−0.56	[49]
40	Quercetin-3-*O*-pentosylhexoside isomer 2 *	8.46	595.1317 [M − H]^−^	596.1387	595, 301, 300, 271, 179, 151	C_26_H_28_O_16_	−1.63	[49]
41	Procyanidin B dimer isomer 5 *	8.74	577.1362 [M − H]^−^	578.1435	577, 451, 425, 407, 289, 161, 125	C_30_H_26_O_12_	−1.77	[45]
42	Procyanidin B trimer isomer 8 *	8.77	865.1990 [M − H]^−^	866.2063	865, 713, 695, 577, 543, 451, 425, 287, 125	C_45_H_38_O_18_	−0.53	[45]
43	Rutin	8.85	609.1456 [M − H]^−^	610.1528	609, 343, 301, 300, 271, 255, 151	C_27_H_30_O_16_	0.96	[49]
44	Kaempferol-3-*O*-hexosylpentoside *	9.07	579.1359 [M − H]^−^	580.1432	579, 285, 284, 255	C_26_H_28_O_15_	−0.72	[49]
45	Hesperidin	9.20	609.1474 [M − H]^−^	610.1549	609, 301, 300, 255	C_28_H_34_O_15_	3.01	[25]
46	Quercetin-3-*O*-β-D-glucoside	9.24	463.0890 [M − H]^−^	464.0963	463, 301, 300, 271, 255, 243, 211, 163, 151	C_21_H_20_O_12_	−1.74	[50]
47	Phloretin-*C*-diglycoside *	9.33	597.1844 [M − H]^−^	598.1915	417, 387, 357, 345, 315, 239, 209	C_27_H_34_O_15_	−2.89	[51]
48	Procyanidin B trimer isomer 9 *	9.37	865.1997 [M − H]^−^	866.2070	865, 713, 695, 577, 543, 451, 425, 287, 125	C_45_H_38_O_18_	−1.35	[45]
49	Procyanidin B tetramer isomer 7 *	9.46	1153.2618 [M − H]^−^	1154.2691	1027, 1001, 983, 865, 577, 575, 449, 423, 413, 289, 287, 125	C_60_H_50_O_24_	0.07	[45]
50	Kaempferol-3-*O*-rutinoside	9.89	593.1511 [M − H]^−^	594.1584	593, 285, 284, 255, 227, 151, 107	C_27_H_30_O_15_	0.08	[44]
51	Kaempferol-3-*O*-β-D-glucoside	10.08	447.0948 [M − H]^−^	448.1020	447, 285, 284, 255, 227	C_21_H_20_O_11_	3.08	[44]
52	Quercetin pentoside *	10.28	433.0766 [M − H]^−^	434.0839	433, 301, 300, 271, 255, 227, 151	C_20_H_18_O_11_	2.32	[44]
53	Kaempferol hexoside isomer 1 *	10.34	447.0941 [M − H]^−^	448.1017	447, 285, 284, 255, 227, 211, 151	C_21_H_20_O_11_	2.37	[44]
54	Kaempferol deoxyhexosylhexoside *	10.58	593.1505 [M − H]^−^	594.1580	593, 285, 284	C_27_H_30_O_15_	0.74	[44]
55	Isorhamnetin-3-*O*-rutinoside *	10.72	623.1611 [M − H]^−^	624.1684	315, 300, 151	C_28_H_32_O_16_	1.09	[52]
56	Kaempferol hexoside isomer 2 *	10.83	447.0927 [M − H]^−^	448.1000	447, 285, 284, 255, 227, 151	C_21_H_20_O_11_	−1.39	[44]
57	Isorhamnetin hexoside *	11.25	477.1052 [M − H]^−^	478.1124	477, 315, 314, 299, 271, 257, 243, 179, 151, 107	C_22_H_22_O_12_	−2.63	[50]
58	(epi)Catechin-ethyl trimer *	11.67	893.2313 [M − H]^−^	894.2371	893, 603, 577, 451, 407, 315, 289, 125	C_47_H_42_O_18_	−2.24	[45]
59	Luteolin *	12.12	285.0416 [M − H]^−^	286.0477	285, 199, 175, 151, 133, 121	C_15_H_10_O_6_	−5.96	[50]
	*Non-flavonoid phenolic compounds and derivatives*							
60	Protocatechuic acid hexoside *	1.31	315.0722 [M − H]^−^	316.0794	315, 153, 152, 123, 109, 108	C_13_H_16_O_9_	−1.90	[50]
61	Hydroxytyrosol hexoside	1.36	315.1095 [M − H]^−^	316.1169	315, 153, 123, 108	C_14_H_20_O_8_	−3.31	[53]
62	Hydroxybenzoic acid hexoside *	1.57	299.0792 [M − H]^−^	300.0865	137	C_13_H_16_O_8_	−6.75	[40]
63	Leonuriside A *	1.62	331.1039 [M − H]^−^	332.1114	169, 153, 125	C_14_H_20_O_9_	−1.95	[54]
64	Caffeoylsucrose isomer 1 *	1.70	503.1417 [M − H]^−^	504.1492	503, 341, 281, 161	C_21_H_28_O_14_	1.70	[55]
65	Hydroxytyrosol hexosylpentoside *	1.75	447.1506 [M − H]^−^	448.1579	153, 123	C_19_H_28_O_12_	0.47	[56]
66	Vanillic acid hexoside isomer 1 *	1.96	329.0892 [M − H]^−^	330.0966	329, 167, 152, 123, 108	C_14_H_18_O_9_	−4.71	[57]
67	Dihydroxybenzoic acid pentoside isomer 1 *	2.01	285.0629 [M − H]^−^	286.0700	285, 152, 108	C_12_H_14_O_8_	−4.09	[58]
68	Caffeoylsucrose isomer 2 *	2.21	503.1409 [M − H]^−^	504.1482	503, 341, 281, 179, 161, 135	C_21_H_28_O_14_	−0.13	[55]
69	Caffeic acid hexoside isomer 1 *	2.34	341.0887 [M − H]^−^	342.0959	341, 179, 161, 135	C_15_H_18_O_9_	1.88	[43]
70	Dihydrocoumaroyl hexoside (3-(2-hydroxyphenyl)-propanoic acid hexose or dihydromelilotoside)	2.40	327.1086 [M − H]^−^	328.1158	165, 147	C_15_H_20_O_8_	−0.09	[59]
71	Syringic acid hexoside	2.78	359.0988 [M − H]^−^	360.1061	197, 182, 167, 153, 138, 123	C_15_H_20_O_10_	1.38	[57]
72	Caffeic acid hexoside isomer 2 *	2.95	341.0880 [M − H]^−^	342.0954	341, 179, 161, 135	C_15_H_18_O_9_	0.18	[43]
73	Vanillic acid hexoside isomer 2 *	3.05	329.0882 [M − H]^−^	330.0955	329, 167, 123	C_14_H_18_O_9_	−1.18	[57]
74	Dihydroxybenzoic acid pentoside isomer 2 *	3.18	285.0621 [M − H]^−^	286.0695	285, 153, 152, 109, 108	C_12_H_14_O_8_	−2.06	[58]
75	Coniferin *	3.35	387.1296 [M + COOH]^−^	342.1315	343, 180, 179, 164	C_16_H_22_O_8_	0.02	[60]
76	Caffeic acid hexoside isomer 3 *	3.39	341.0886 [M − H]^−^	342.0959	341, 179, 161, 135	C_15_H_18_O_9_	1.79	[43]
77	*p-*Coumaric acid hexoside	3.44	325.0931 [M − H]^−^	326.1004	163, 145, 117	C_15_H_18_O_8_	−0.77	[25]
78	Caffeic acid	3.70	179.0352 [M − H]^−^	180.0425	179, 135, 134, 107	C_9_H_8_O_4_	−1.63	[15]
79	Vanillic acid hexoside isomer 3 *	3.91	329.0886 [M − H]^−^	330.0959	329, 167, 123	C_14_H_18_O_9_	−2.48	[57]
80	Caffeoylquinic acid (Chlorogenic acid)	4.17	353.0879 [M − H]^−^	354.0953	191, 179, 161, 135	C_16_H_18_O_9_	−0.65	[50]
81	Ferulic acid hexoside	4.41	355.1039 [M − H]^−^	356.1112	193, 175, 134	C_16_H_20_O_9_	−0.30	[25]
82	Methylsyringin*	5.86	385.1510 [M − H]^−^	386.1581	223, 179, 161	C_18_H_26_O_9_	−1.20	[41]
83	Caffeoylshikimic acid*	6.47	335.0781 [M − H]^−^	336.0854	179, 161, 135	C_16_H_16_O_8_	−2.59	[61]
84	Ferulic acid	6.73	193.0508 [M − H]^−^	194.0581	134	C_10_H_10_O_4_	−0.97	[62]
85	Phloroacetophenone 6′-[xylosyl-(1→6)-glucoside] *	7.38	489.1617 [M − H]^−^	490.1691	168	C_21_H_30_O_13_	−1.03	[41]
86	Caffeoyltyramine isomer 1 *	8.12	298.1094 [M − H]^−^	299.1167	298, 178, 135	C_17_H_17_NO_4_	−0.23	[15]
87	Pinoresinol hexoside *	9.73	519.1864 [M − H]^−^	520.1937	357, 342, 311, 151, 136	C_26_H_32_O_11_	1.40	[63]
88	Pinoresinol *	9.76	357.1339 [M − H]^−^	358.1411	342, 311, 151, 136	C_20_H_22_O_6_	1.57	[64]
89	Caffeoyltyramine isomer 2 *	10.49	298.1100 [M − H]^−^	299.1172	298, 178, 161, 135	C_17_H_17_NO_4_	1.43	[15]
90	Syringaresinol-*O*-β-D-glucopyranoside *	10.72	579.2076 [M − H]^−^	580.2148	417, 181	C_28_H_36_O_13_	1.37	[65]
91	Caffeic acid ethyl ester *	10.89	207.0657 [M − H]^−^	208.0729	179, 161, 135, 133	C_11_H_12_O_4_	3.11	[66]
92	Lavandulifolioside *	10.98	755.2463 [M − H]^−^	756.2477	593	C_34_H_44_O_19_	−8.47	[67]
93	*N-*Feruloyltyramine *	11.93	312.1241 [M − H]^−^	313.1314	312, 297, 178, 135	C_18_H_19_NO_4_	0.12	[68]
94	4-Hydroxyphenyl-hexanoic acid *	12.43	207.1035 [M − H]^−^	208.1109	193, 149, 135, 119	C_12_H_16_O_3_	−4.70	[69]
95	*p-*Coumaric acid methyl ester	12.66	177.0917 [M − H]^−^	178.0991	163, 145	C_11_H_14_O_2_	1.72	[25]
96	Verimol H *	12.97	327.1612 [M − H]^−^	328.1675	327, 165, 145	C_20_H_24_O_4_	−2.80	[41]
97	*p-*Decycloxybenzoic acid *	13.42	277.1816 [M − H]^−^	278.1889	233, 205	C_17_H_26_O_3_	−2.46	[70]
	*Alkaloids*							
98	Bakankoside *	2.18	356.1363 [M − H]^−^	357.1435	195, 194, 123	C_16_H_23_NO_8_	−3.06	[41]
99	Isoboldine	5.99	326.1407 [M − H]^−^	327.1481	326, 312, 311, 297, 296, 281, 268, 267, 253, 252, 29, 225, 197	C_19_H_21_NO_4_	−3.23	[71]
100	Damascenine *	13.10	194.0831 [M − H]^−^	195.0904	179, 149	C_10_H_13_NO_3_	−4.58	[72]
	*Annonaceous acetogenins*							
101	Unknown acetogenin 1 *	13.31	627.4472 [M − H]^−^	628.4545	565, 501, 197, 127	C_35_H_64_O_9_	0.88	[73]
102	Unknown acetogenin 2 *	13.40	671.4750 [M − H]^−^	672.4823	609, 545, 127	C_37_H_68_O_10_	−1.52	[73]
103	Unknown acetogenin 3 *	13.49	655.4792 [M − H]^−^	656.4864	637, 611, 127	C_37_H_68_O_9_	−0.16	[73]
104	Unknown acetogenin 4 *	13.53	627.4488 [M − H]^−^	628.4564	609, 583, 565, 501, 127	C_35_H_64_O_9_	−2.23	[73]
105	Unknown acetogenin 5 *	13.66	627.4481 [M − H]^−^	628.4554	609, 583, 565, 501, 127	C_35_H_64_O_9_	−0.58	[73]
106	Unknown acetogenin 6 *	13.83	653.4630 [M − H]^−^	654.4707	635, 609, 599, 591, 581, 527, 491, 127	C_37_H_66_O_9_	−0.15	[73]
107	Unknown acetogenin 7 *	13.88	609.4377 [M − H]^−^	610.4463	547, 483, 197, 127	C_35_H_62_O_8_	−3.01	[73]
108	Unknown acetogenin 8 *	13.89	655.4789 [M − H]^−^	656.4863	637, 619, 611, 593, 583, 565, 529, 197, 127	C_37_H_68_O_9_	−0.61	[73]
109	Unknown acetogenin 9 *	13.98	611.4526 [M − H]^−^	612.4604	593, 575, 567, 549, 197, 127	C_35_H_64_O_8_	−0.48	[73]
110	Unknown acetogenin 10 *	14.09	637.4694 [M − H]^−^	638.4766	619, 593, 575, 525, 511, 493, 127	C_37_H_66_O_8_	−1.33	[73]
111	Unknown acetogenin 11 *	14.14	609.4370 [M − H]^−^	610.4489	591, 573, 547, 483, 465, 127	C_35_H_62_O_8_	−7.26	[73]
112	Unknown acetogenin 12 *	14.22	639.4852 [M − H]^−^	640.4923	621, 603, 595, 577, 527, 513, 127	C_37_H_68_O_8_	−1.38	[73]
113	Unknown acetogenin 13 *	14.35	637.4694 [M − H]^−^	638.4766	619, 593, 575, 511, 197, 127	C_37_H_66_O_8_	−1.34	[73]
114	Unknown acetogenin 14 *	14.53	621.4720 [M − H]^−^	622.4821	577, 509, 197, 127	C_37_H_66_O_7_	−2.01	[73]
115	Unknown acetogenin 15 *	14.57	595.4589 [M − H]^−^	596.4661	577, 533, 197, 127	C_35_H_64_O_7_	−1.57	[73]
116	Unknown acetogenin 16 *	14.57	623.4892 [M − H]^−^	624.4965	579, 561, 127	C_37_H_68_O_7_	0.05	[73]
117	Unknown acetogenin 17 *	14.66	619.4587 [M − H]^−^	620.4675	601, 575, 507, 127	C_37_H_64_O_7_	−3.75	[73]
118	Unknown acetogenin 18 *	14.79	623.4899 [M − H]^−^	624.4972	561, 497, 127	C_37_H_68_O_7_	−1.12	[73]
	*Fatty acids and derivatives*							
119	*n-*Hydroxyhexanoic acid hexoside *	1.79	293.1244 [M − H]^−^	294.1316	173, 131	C_12_H_22_O_8_	−0.34	[51]
120	6E-Octene-2,4-diynoic acid *	2.35	133.0298 [M − H]^−^	134.0371	133, 115	C_8_H_6_O_2_	−2.03	[74]
121	Prenyl arabinosyl-(1→6)-glucoside *	2.40	379.1615 [M − H]^−^	380.1688	191, 149, 131	C_16_H_28_O_10_	−1.56	[41]
122	Pentyl-pentosylhexoside *	3.18	381.1771 [M − H]^−^	382.1844	249, 161, 113, 101	C_16_H_30_O_10_	−1.26	[51]
123	1-(3-Methylbutanoyl)-6-apiosylglucose *	3.70	395.1555 [M − H]^−^	396.1629	395, 249, 163, 161, 113, 101	C_16_H_28_O_11_	0.78	[41]
124	8:1 + 2O fatty acyl hexoside isomer 1 *	4.22	321.1559 [M − H]^−^	322.1628	321, 159	C_14_H_26_O_8_	−1.18	[75]
125	8:1 + 2O fatty acyl hexoside isomer 2 *	4.56	321.1555 [M − H]^−^	322.1628	159	C_14_H_26_O_8_	0.29	[75]
126	8:1 + 2O fatty acyl hexoside isomer 3 *	6.51	321.1556 [M − H]^−^	322.1629	159	C_14_H_26_O_8_	−0.35	[75]
127	9,12,13-Trihydroxy-octadecadienoic acid *	12.32	327.2191 [M − H]^−^	328.2265	229, 211, 197, 183, 171	C_18_H_32_O_5_	−4.61	[51]
128	11-Hydroperoxy-octadecatrienoic acid *	13.05	309.2080 [M − H]^−^	310.2156	291, 209, 185, 121	C_18_H_30_O_4_	−3.77	[65]
	*Other compounds*							
129	L-Arginine *	0.58	173.1053 [M − H]^−^	174.1126	131, 127	C_6_H_14_N_4_O_2_	−5.57	[71]
130	Glucuronic or galacturonic acid *	0.62	193.0365 [M − H]^−^	194.0437	177, 130, 113, 103	C_6_H_10_O_7_	−5.52	[71]
131	Gluconic acid	0.62	195.0513 [M − H]^−^	196.0586	195, 177, 129	C_6_H_12_O_7_	−1.74	[35]
132	Pantothenic acid hexoside *	1.27	380.1572 [M − H]^−^	381.1644	380, 218, 146	C_15_H_27_NO_10_	−2.31	[46]
133	Dehydrophaseic acid hexoside *	2.92	443.1919 [M − H]^−^	444.1992	281, 237, 219, 189, 161, 153, 143, 119	C_21_H_32_O_10_	0.88	[76]
134	Benzyl-pentosylhexoside *	2.92	401.1454 [M − H]^−^	402.1528	269, 161, 113, 101	C_18_H_26_O_10_	−0.51	[51]
135	Methylbenzoic acid *	3.74	135.0454 [M − H]^−^	136.0527	135, 134, 119, 117, 107	C_8_H_8_O_2_	−1.91	[71]
136	1-Hexanol arabinosylglucoside *	5.95	441.1976 [M + COOH]^−^	396.1992	395, 263	C_17_H_32_O_10_	0.81	[41]
137	Roseoside *	5.00	431.1921 [M + COOH]^−^	386.1948	385, 153	C_19_H_30_O_8_	−1.80	[35]
138	(1S,2S,4R,8S)-*p-*Menthane-1,2,8,9-tetrol-2-glucoside *	6.11	359.0988 [M − H]^−^	366.1888	203, 157	C_16_H_30_O_9_	0.42	[41]
139	Abscisic acid *	11.41	263.1286 [M − H]^−^	264.1359	219, 204, 203, 199, 173, 171, 163, 153, 149, 139, 137	C_15_H_20_O_4_	1.00	[71]

* Compounds reported for the first time in the araticum pulp.

**Table 3:** Specific fragmentation pathways of tentatively annotated annonaceous acetogenins found in the araticum pulp extract by UHPLC-ESI-QTOF-MS/MS under negative ion mode.

Tentatively Annotated Annonaceous Acetogenins	Precursor Molecular Ion ([M − H]^−^) (*m*/*z*)	MS/MS Fragment Ions (*m*/*z*)	Specific Fragmentation Pathway
Unknown acetogenin 1	627.4472	565.4436 [M-H-CO_2_-H_2_O]^−^	Fragment ion resulting from the successive loss of one water molecule and one CO_2_ molecule
		501.4063 [M-H-7H_2_O]^−^	Fragment ion resulting from the successive loss of seven water molecules
		197.1874 [C_13_H_25_O]^−^	Fragment ion produced by cleavage in the alpha of the first hydroxyl group starting from the methyl extremity
		127.0396 [C_6_H_7_O_3_]^−^	Fragment ion referring to the tetrahydrofuran ring
Unknown acetogenin 2	671.4750	609.4686 [M-H-CO_2_-H_2_O]^−^	Fragment ion resulting from the successive loss of one water molecule and one CO_2_ molecule
		545.4426 [M-H-7H_2_O]^−^	Fragment ion resulting from the successive loss of seven water molecules
		127.0416 [C_6_H_7_O_3_]^−^	Fragment ion referring to the tetrahydrofuran ring
Unknown acetogenin 3	655.4792	637.4572 [M-H-H_2_O]^−^	Fragment ion resulting from the loss of one water molecule
		611.4484 [M-H-CO_2_]^−^	Fragment ion resulting from the loss of one CO_2_ molecule
		127.0395 [C_6_H_7_O_3_]^−^	Fragment ion referring to the tetrahydrofuran ring
Unknown acetogenin 4	627.4488	609.4336 [M-H-H_2_O]^−^	Fragment ion resulting from the loss of one water molecule
		583.3684 [M-H-CO_2_]^−^	Fragment ion resulting from the loss of one CO_2_ molecule
		565.4461 [M-H-CO_2_-H_2_O]^−^	Fragment ion resulting from the successive loss of one water molecule and one CO_2_ molecule
		501.4140 [M-H-7H_2_O]^−^	Fragment ion resulting from the successive loss of seven water molecules
		127.0395 [C_6_H_7_O_3_]^−^	Fragment ion referring to the tetrahydrofuran ring
Unknown acetogenin 5	627.4481	609.4395 [M-H-H_2_O]^−^	Fragment ion resulting from the loss of one water molecule
		583.3489 [M-H-CO_2_]^−^	Fragment ion resulting from the loss of one CO_2_ molecule
		565.4556 [M-H-CO_2_-H_2_O]^−^	Fragment ion resulting from the successive loss of one water molecule and one CO_2_ molecule
		501.4124 [M-H-7H_2_O]^−^	Fragment ion resulting from the successive loss of seven water molecules
		127.0398 [C_6_H_7_O_3_]^−^	Fragment ion referring to the tetrahydrofuran ring
Unknown acetogenin 6	653.4630	635.4531 [M-H-H_2_O]^−^	Fragment ion resulting from the loss of one water molecule
		609.4309 [M-H-CO_2_]^−^	Fragment ion resulting from the loss of one CO_2_ molecule
		599.4428 [M-H-3H_2_O]^−^	Fragment ion resulting from the successive loss of three water molecules
		591.4620 [M-H-CO_2_-H_2_O]^−^	Fragment ion resulting from the successive loss of one water molecule and one CO_2_ molecule
		581.4394 [M-H-4H_2_O]^−^	Fragment ion resulting from the successive loss of four water molecules
		527.4343 [M-H-7H_2_O]^−^	Fragment ion resulting from the successive loss of seven water molecules
		491.4131 [M-H-9H_2_O]^−^	Fragment ion resulting from the successive loss of nine water molecules
		127.0403 [C_6_H_7_O_3_]^−^	Fragment ion referring to the tetrahydrofuran ring
Unknown acetogenin 7	609.4377	547.4355 [M-H-CO_2_-H_2_O]^−^	Fragment ion resulting from the successive loss of one water molecule and one CO_2_ molecule
		483.4054 [M-H-7H_2_O]^−^	Fragment ion resulting from the successive loss of seven water molecules
		197.1920 [C_13_H_25_O]^−^	Fragment ion produced by cleavage in the alpha of the first hydroxyl group starting from the methyl extremity
		127.0402 [C_6_H_7_O_3_]^−^	Fragment ion referring to the tetrahydrofuran ring
Unknown acetogenin 8	655.4789	637.4616 [M-H-H_2_O]^−^	Fragment ion resulting from the loss of one water molecule
		619.4591 [M-H-2H_2_O]^−^	Fragment ion resulting from the successive loss of two water molecules
		611.4513 [M-H-CO_2_]^−^	Fragment ion resulting from the loss of one CO_2_ molecule
		593.4770 [M-H-CO_2_-H_2_O]^−^	Fragment ion resulting from the successive loss of one water molecule and one CO_2_ molecule
		583.4575 [M-H-4H_2_O]^−^	Fragment ion resulting from the successive loss of four water molecules
		565.4437 [M-H-5H_2_O]^−^	Fragment ion resulting from the successive loss of five water molecules
		529.4359 [M-H-7H_2_O]^−^	Fragment ion resulting from the successive loss of seven water molecules
		197.1921 [C_13_H_25_O]^−^	Fragment ion produced by cleavage in the alpha of the first hydroxyl group starting from the methyl extremity
		127.0405 [C_6_H_7_O_3_]^−^	Fragment ion referring to the tetrahydrofuran ring
Unknown acetogenin 9	611.4526	593.4368 [M-H-H_2_O]^−^	Fragment ion resulting from the loss of one water molecule
		575.4350 [M-H-2H_2_O]^−^	Fragment ion resulting from the successive loss of two water molecules
		567.4716 [M-H-CO_2_]^−^	Fragment ion resulting from the loss of one CO_2_ molecule
		549.4527 [M-H-CO_2_-H_2_O]^−^	Fragment ion resulting from the successive loss of one water molecule and one CO_2_ molecule
		197.1918 [C_13_H_25_O]^−^	Fragment ion produced by cleavage in the alpha of the first hydroxyl group starting from the methyl extremity
		127.0400 [C_6_H_7_O_3_]^−^	Fragment ion referring to the tetrahydrofuran ring
Unknown acetogenin 10	637.4694	619.4571 [M-H-H_2_O]^−^	Fragment ion resulting from the loss of one water molecule
		593.4720 [M-H-CO_2_]^−^	Fragment ion resulting from the loss of one CO_2_ molecule
		575.4471 [M-H-CO_2_-H_2_O]^−^	Fragment ion resulting from the successive loss of one water molecule and one CO_2_ molecule
		525.4318 [M-C_6_H_8_O_2_]^−^	Fragment ion resulting from the loss of the terminal γ-lactone ring
		511.4320 [M-H-7H_2_O]^−^	Fragment ion resulting from the successive loss of seven water molecules
		493.4218 [M-H-8H_2_O]^−^	Fragment ion resulting from the successive loss of eight water molecules
		127.0401 [C_6_H_7_O_3_]^−^	Fragment ion referring to the tetrahydrofuran ring
Unknown acetogenin 11	609.4370	591.4309 [M-H-H_2_O]^−^	Fragment ion resulting from the loss of one water molecule
		573.4091 [M-H-2H_2_O]^−^	Fragment ion resulting from the successive loss of two water molecules
		547.4392 [M-H-CO_2_-H_2_O]^−^	Fragment ion resulting from the successive loss of one water molecule and one CO_2_ molecule
		483.4055 [M-H-7H_2_O]^−^	Fragment ion resulting from the successive loss of seven water molecules
		465.3989 [M-H-8H_2_O]^−^	Fragment ion resulting from the successive loss of eight water molecules
		127.0401 [C_6_H_7_O_3_]^−^	Fragment ion referring to the tetrahydrofuran ring
Unknown acetogenin 12	639.4852	621.4720 [M-H-H_2_O]^−^	Fragment ion resulting from the loss of one water molecule
		603.4564 [M-H-2H_2_O]^−^	Fragment ion resulting from the successive loss of two water molecules
		595.4555 [M-H-CO_2_]^−^	Fragment ion resulting from the loss of one CO_2_ molecule
		577.4825 [M-H-CO_2_-H_2_O]^−^	Fragment ion resulting from the successive loss of one water molecule and one CO_2_ molecule
		527.4503 [M-C_6_H_8_O_2_]^−^	Fragment ion resulting from the loss of the terminal γ-lactone ring
		513.4373 [M-H-7H_2_O]^−^	Fragment ion resulting from the successive loss of seven water molecules
		127.0406 [C_6_H_7_O_3_]^−^	Fragment ion referring to the tetrahydrofuran ring
Unknown acetogenin 13	637.4694	619.4532 [M-H-H_2_O]^−^	Fragment ion resulting from the loss of one water molecule
		593.4496 [M-H-CO_2_]^−^	Fragment ion resulting from the loss of one CO_2_ molecule
		575.4707 [M-H-CO_2_-H_2_O]^−^	Fragment ion resulting from the successive loss of one water molecule and one CO_2_ molecule
		511.4383 [M-H-7H_2_O]^−^	Fragment ion resulting from the successive loss of seven water molecules
		197.1955 [C_13_H_25_O]^−^	Fragment ion produced by cleavage in the alpha of the first hydroxyl group starting from the methyl extremity
		127.0402 [C_6_H_7_O_3_]^−^	Fragment ion referring to the tetrahydrofuran ring
Unknown acetogenin 14	621.4720	577.4801 [M-H-CO_2_]^−^	Fragment ion resulting from the loss of one CO_2_ molecule
		509.4241 [M-C_6_H_8_O_2_]^−^	Fragment ion resulting from the loss of the terminal γ-lactone ring
		197.1937 [C_13_H_25_O]^−^	Fragment ion produced by cleavage in the alpha of the first hydroxyl group starting from the methyl extremity
		127.0390 [C_6_H_7_O_3_]^−^	Fragment ion referring to the tetrahydrofuran ring
Unknown acetogenin 15	595.4589	577.4471 [M-H-H_2_O]^−^	Fragment ion resulting from the loss of one water molecule
		533.4516 [M-H-CO_2_-H_2_O]^−^	Fragment ion resulting from the successive loss of one water molecule and one CO_2_ molecule
		197.1914 [C_13_H_25_O]^−^	Fragment ion produced by cleavage in the alpha of the first hydroxyl group starting from the methyl extremity
		127.0397 [C_6_H_7_O_3_]^−^	Fragment ion referring to the tetrahydrofuran ring
Unknown acetogenin 16	623.4892	579.4922 [M-H-CO_2_]^−^	Fragment ion resulting from the loss of one CO_2_ molecule
		561.9243 [M-H-CO_2_-H_2_O]^−^	Fragment ion resulting from the successive loss of one water molecule and one CO_2_ molecule
		127.0406 [C_6_H_7_O_3_]^−^	Fragment ion referring to the tetrahydrofuran ring
Unknown acetogenin 17	619.4587	601.4414 [M-H-H_2_O]^−^	Fragment ion resulting from the loss of one water molecule
		575.2319 [M-H-CO_2_]^−^	Fragment ion resulting from the loss of one CO_2_ molecule
		507.2350 [M-C_6_H_8_O_2_]^−^	Fragment ion resulting from the loss of the terminal γ-lactone ring
		127.0399 [C_6_H_7_O_3_]^−^	Fragment ion referring to the tetrahydrofuran ring
Unknown acetogenin 18	623.4899	561.4874 [M-H-CO_2_-H_2_O]^−^	Fragment ion resulting from the successive loss of one water molecule and one CO_2_ molecule
		497.4376 [M-H-7H_2_O]^−^	Fragment ion resulting from the successive loss of seven water molecules
		127.0404 [C_6_H_7_O_3_]^−^	Fragment ion referring to the tetrahydrofuran ring

## Data Availability

The authors confirm that the data supporting the findings of this study are available within the article.
